# Clinical, laboratory data and inflammatory biomarkers at baseline as early discharge predictors in hospitalized SARS-CoV-2 infected patients

**DOI:** 10.1371/journal.pone.0269875

**Published:** 2022-07-14

**Authors:** María Trujillo-Rodriguez, Esperanza Muñoz-Muela, Ana Serna-Gallego, Juan Manuel Praena-Fernández, Alberto Pérez-Gómez, Carmen Gasca-Capote, Joana Vitallé, Joaquim Peraire, Zaira R. Palacios-Baena, Jorge Julio Cabrera, Ezequiel Ruiz-Mateos, Eva Poveda, Luis Eduardo López-Cortés, Anna Rull, Alicia Gutierrez-Valencia, Luis Fernando López-Cortés

**Affiliations:** 1 Clinical Unit of Infectious Diseases, Microbiology and Preventive Medicine, Institute of Biomedicine of Seville (IBiS), Virgen del Rocío University Hospital, CSIC, University of Seville, Seville, Spain; 2 Department of Biostatistics, Faculty of Medicine, University of Granada, Granada, Spain; 3 Hospital Universitari de Tarragona Joan XXIII (HJ23), Tarragona, Spain; 4 Institut Investigació Sanitària Pere Virgili (IISPV), Tarragona, Spain; 5 CIBER Enfermedades Infecciosas, Instituto de Salud Carlos III, Madrid, Spain; 6 Universitat Rovira i Virgili (URV), Tarragona, Spain; 7 Clinical Unit of Infectious Diseases and Microbiology, Institute of Biomedicine of Seville (IBiS), Virgen del Rocío and Virgen Macarena University Hospitals/CSIC/University of Seville, Seville, Spain; 8 Clinical Unit of Infectious Diseases and Microbiology, Virgen Macarena University Hospital, Seville, Spain; 9 Group of Virology and Pathogenesis, Galicia Sur Health Research Institute (IIS Galicia Sur) Complexo Hospitalario Universitario de Vigo, SERGAS-UVigo, Vigo, Spain; 10 Microbiology Service, Galicia Sur Health Research Institute (IIS Galicia Sur), Complexo Hospitalario Universitario de Vigo, SERGAS-UVigo, Vigo, Spain; Tanta University Faculty of Medicine, EGYPT

## Abstract

**Background:**

The SARS-CoV-2 pandemic has overwhelmed hospital services due to the rapid transmission of the virus and its severity in a high percentage of cases. Having tools to predict which patients can be safely early discharged would help to improve this situation.

**Methods:**

Patients confirmed as SARS-CoV-2 infection from four Spanish hospitals. Clinical, demographic, laboratory data and plasma samples were collected at admission. The patients were classified into mild and severe/critical groups according to 4-point ordinal categories based on oxygen therapy requirements. Logistic regression models were performed in mild patients with only clinical and routine laboratory parameters and adding plasma pro-inflammatory cytokine levels to predict both early discharge and worsening.

**Results:**

333 patients were included. At admission, 307 patients were classified as mild patients. Age, oxygen saturation, Lactate Dehydrogenase, D-dimers, neutrophil-lymphocyte ratio (NLR), and oral corticosteroids treatment were predictors of early discharge (area under curve (AUC), 0.786; sensitivity (SE) 68.5%; specificity (S), 74.5%; positive predictive value (PPV), 74.4%; and negative predictive value (NPV), 68.9%). When cytokines were included, lower interferon-γ-inducible protein 10 and higher Interleukin 1 beta levels were associated with early discharge (AUC, 0.819; SE, 91.7%; S, 56.6%; PPV, 69.3%; and NPV, 86.5%). The model to predict worsening included male sex, oxygen saturation, no corticosteroids treatment, C-reactive protein and Nod-like receptor as independent factors (AUC, 0.903; SE, 97.1%; S, 68.8%; PPV, 30.4%; and NPV, 99.4%). The model was slightly improved by including the determinations of interleukine-8, Macrophage inflammatory protein-1 beta and soluble IL-2Rα (CD25) (AUC, 0.952; SE, 97.1%; S, 98.1%; PPV, 82.7%; and NPV, 99.6%).

**Conclusions:**

Clinical and routine laboratory data at admission strongly predict non-worsening during the first two weeks; therefore, these variables could help identify those patients who do not need a long hospitalization and improve hospital overcrowding. Determination of pro-inflammatory cytokines moderately improves these predictive capacities.

## Introduction

The current SARS-CoV-2 pandemic have overwhelmed many countries’ health services [1]. Around 20% of patients have pulmonary involvement that requires hospital admission instead of home care, as in milder cases [2, 3]. Many of them remain in stable clinical condition during hospital stay, respond to conservative treatment, and could be discharged early. By contrast, other patients progress to respiratory failure requiring more intense ventilatory support, of whom a non-negligible percentage will die [3]. The uncertainty of which patients will worsen frequently makes hospitalization longer. A model that reliably predicts which patients will not progress would facilitate early hospital discharges and relieve inpatient hospital care.

Several factors have been identified as predictors of the severity or progression of SARS-CoV-2 infection, such as older age and the presence of comorbidities such as hypertension, obesity, chronic obstructive pulmonary disease, diabetes, and cardiovascular disease [4]. In addition, clinical data and laboratory parameters such as lymphopenia, high plasma levels of lactate dehydrogenase (LDH), C-reactive protein (CRP), ferritin and D-dimer have been associated with a worse evolution and/or mortality. However, most of these studies do not provide the positive and negative predictive value of their model, making it difficult to know the true predictive capacity of these parameters [5–11].

An association has been reported between high plasma levels of some cytokines, such as interleukin (IL)-6, soluble IL-2 receptor (sCD25), IL-10, and tumor necrosis factor α (TNF-α) [12, 13], and other inflammatory mediators with an unfavorable prognosis and the need for intensive care unit (ICU) admission [14–18].

The present study aims to investigate whether the combination of clinical, routine laboratory data and plasma cytokine concentrations at baseline can predict with certainty which patients could be safely discharged early from the hospital.

## Materials and methods

### Study design and participants

The study was a prospective multicenter study carried out in four Spanish hospitals: Joan XXIII Hospital, Tarragona, Virgen Macarena University Hospital, Seville, Virgen del Rocio University Hospital, Seville, and Alvaro Cunqueiro Hospital Vigo (Cohort COVID-19 of the Galicia Sur Health Research Institute), where all patients older than 18 years old with confirmed SARS-CoV-2 infection, radiologic infiltrates on chest X-ray and/or severe hypoxemia (SpO_2_ <94%) admitted to the hospital were sequentially included in the study from March to September 2020. The only exclusion criteria were the use of corticosteroids equivalent to >5 mg/day of prednisone and/or anti-inflammatory biologic drugs prior to hospitalization.

The diagnosis was made by viral gene detection using nasopharyngeal swab samples and real-time polymerase chain reaction (RT-PCR). Viral RNA extraction was carried out by an automated nucleic acid purification platform (The Maxwell® RSC Instrument). The assay contains primers and probes targeting three regions of the SARS-CoV-2; orf1ab; spike (S) gene; nucleocapsid (N) gene and RNase P gene as control. Plasma samples from all patients were collected at admission. A standard checklist was used to record information extracted from electronic medical records, including demographic variables, clinical, laboratory data on admission and during the hospitalization.

The primary endpoint was to assess whether the determination of pro-inflammatory cytokines at admission, together with clinical and routine analytical parameters, could predict a good short-term evolution and facilitate an early discharge, defined as that within the first week after admission.

The discharge criteria were clinical stability, apirexia, and stable oximetry without requiring increased oxygen intake for at least 48 hours, as well as improvement of analytical inflammatory reactants.

Patients were classified according to 4-point ordinal categories based on WHO R&D blueprint and COVID-19: mild, oxygen therapy with mask or nasal prongs; severe, high-flow oxygen requirement or non-invasive ventilation; critical, mechanical ventilation or extracorporeal membrane oxygenation, and death [19].

### Ethics approval statement

The study was conducted according to Good Clinical Practice principles after being approved by the Ethics Committee for Clinical Research of Virgen Macarena and Virgen del Rocio University Hospitals, Seville, Spain (internal code, 0767-N-20) and the National Health Authority. All patients who agreed to participate provided oral or written informed consent before undergoing any study-related procedures.

### Laboratory parameters

Hematological and biochemical profiles were evaluated in all patients upon admission to each hospital. Among them, CRP was determined by an immunoturbidimetric method (Cobas 701; Roche Diagnostics, Mannheim, Germany), D-dimer by an automated latex-enhanced immunoassay (HemosIL D-Dimer HS 500, Instrumentation Laboratory, Bedford, Massachusetts), LDH and ferritin were determined by enzyme-linked immunosorbent assay (ELISA).

### Plasma cytokine concentrations

Plasma cytokine concentrations were measured in aliquot samples obtained at admission and stored at -80°C until analysis. IL-6, IL-8, IL-1β, TNF-α, interferon-gamma (IFN-γ), macrophage inflammatory proteins 1α (MIP-1α) and β (MIP-1β) were analyzed using a multiplex bead-based immunoassay (MILLIPLEX® MAP human high-sensitivity T cell magnetic bead panel). Interferon gamma-induced protein 10 (IP-10) and sCD25 were measured by the Human CXCL10 ELISA kit (Abcam, Cambridge, UK) and the Human Quantikine Immunoassay (R&D Systems), respectively. All samples were analyzed in duplicate and the median values were used as result for statistical analysis, unless the difference between the two determinations was greater than 30%, in which case the determinations were repeated.

### Monocyte immunophenotyping

Peripheral blood mononuclear cells (PBMCs) were isolated using a Ficoll density gradient and stored with fetal bovine serum (FBS) and 10% dimethylsulfoxide (DMSO) in liquid nitrogen until the time of the assay. Cells were stained with a viability marker (LIVE/DEAD Fixable Violet Dead Cell Stain Kit 405nm, Life Technologies) and different fluorochrome-conjugated antibodies for 35 min at RT to assess the expression of surface marker expression. Anti-CD3, anti-CD19, anti-CD20, and anti-CD56 conjugated with V450 were used as a dump channel. Anti-CD14-BV650 and anti-CD16-PeCF594 (BD biosciences, USA) were used to classify different subsets of monocytes. Anti-TLR2-FITC, anti-HLA-DR-BV570, anti-CD40-APC (all BD Biosciences, USA), and anti-TLR4-BV786 (Biolegend, USA) were determined as activation markers. Anti-CX3CR1-PerCPCy5,5, anti-CCR2-BV605, anti-CD49d- BV711, anti-CD142-PE (all Biolegend, USA), anti-CCR5- APC-Cy7, and anti-CD11b-AF700 (all from BD Biosciences, USA) were used as maturation and homing markers. The cellular markers were analyzed by multiparametric flow cytometry using the Fortessa LSR II cytometer (BD Biosciences, Madrid, Spain). A minimum of 1x10^6^ events were acquired per sample. Data were analyzed using Flowjo 9.3.2 software.

### Statistical analysis

The results were expressed as median values and interquartile ranges (IQR) for continuous variables and as numbers and percentages of cases for categorical variables. Categorical variables were compared using the χ^2^ test or Fisher’s exact test. Quantitative variables were analyzed using the Student’s t-test or the Mann–Whitney nonparametric test, according to their distribution. Receiver operating characteristic curve (ROC) analyzes were performed to assess the accuracy of different levels of biomarkers in predicting the events, using the Youden index to choose the best cut-off point. To predict factors associated with early discharge or worsening of the patients, logistic regression models were carried out using clinically relevant and statistically significant variables in the previous bivariate analysis. Continuous variables were squared-root or log-transformed when necessary to satisfy model assumptions. Akaike information criterion (AIC) was used to select the best model, and the Hosmer-Lemeshow statistic and the Nagelkerke pseudo-R^2^ were performed to determine the goodness of fit. Internal validation was performed using two methods: K-Fold and leave-one-out cross-validation. The mean of the correct classification rates was used as a validation measure.

Statistical analyzes were performed using IBM software (SPSS, version 26.0; SPSS, Chicago, IL). Software R (Foundation for Statistical Computing, Vienna, Austria) and GraphPad Prism v 8.0. p values <0.05 were considered significant.

## Results

### Study population

Three hundred thirty-three subjects were included from March to September 2020. The baseline characteristics of the study population are summarized in Table 1. Overall, the median time elapsed between the onset of symptoms and admission was eight days (5−11). All the samples were taken on admission ± 8 hours. Regarding oxygen requirement at admission, 307 patients (92.2%) were classified as mild, 22 (6.6%) as severe, and 4 (1.2%) as critical. Severe and critical patients were analyzed together. We found several differences between the mild and severe/critical groups. The last one was older, had lower peripheral capillary oxygen saturation (SpO_2_), and higher levels of some cytokines at admission. Ferritin was also more elevated in several/critical patients, although without reaching full statistical significance (p = 0.054).

**Table 1:** Baseline characteristics of the patients.

	Overall	Mild	Severe/Critical	
	(n = 333)	(n = 307)	(n = 26)	p
Age (years)	63 (50–75)	63 (49–75)	72 (60–82)	**0.005**
Male sex.	205 (61.5)	193 (62.9)	12 (46.2)	0.093
Comorbidities	243 (73.0)	221 (72.0)	22 (84.6)	0.164
Diabetes mellitus	70 (21.0)	65 (21.2)	5 (19.2)	0.820
Hypertension	149 (44.7)	133 (43.3)	17 (61.5)	0.065
Heart disease	58 (17.4)	53 (17.3)	5 (19.2)	0.788
Chronic pulmonary disease	37 (11.1)	35(11.4)	2 (7.7)	0.569
Cancer	20 (6.0)	19 (6.2)	1 (3.8)	0.634
Symptoms on admission				
Cough	214 (64.3)	201 (65.5)	13 (50.0)	0.068
Fever	198 (59.5)	184 (59.9)	14 (53.8)	0.388
Dyspnea	158 (47.4)	141 (45.9)	17 (65.4)	**0.011**
Diarrhea	80 (24.0)	76 (24.8)	4 (15.4)	0.724
Arthromyalgia	71 (21.3)	66 (21.5)	5 (19.2)	0.248
SpO_2_ (%)	95 (92–97)	95 (92–97)	86 (80–90)	**0.000**
Days of symptoms until sampling	8 (5–11)	8 (5–11)	7 (5–9)	0.126
CRP (mg/L)	25 (7–88)	23 (6–79)	120 (17–234)	**0.001**
LDH (UI/L)	271 (223–344)	267 (222–328)	400 (272–552)	**0.000**
D-dimer (ng/mL)	670 (404–1137)	620 (394–1058)	1091 (729–1732)	**0.001**
Ferritin (ng/mL)	440 (228–976)	419 (221–967)	755 (424–1357)	0.054
Lymphocytes (x 10^9^/L)	1.04 (0.75–1.50)	1.07 (0.76–1.50)	0.8 (0.48–1.20)	**0.019**
Neutrophil (x 10^9^/L)	6.25 (4.10–7.95)	6.07 (3.94–7.81)	7.56 (5.82–9.16)	**0.001**
Monocytes (x 10^9^/L)	0.44 (0.31–0.620)	0.45 (0.32–0.64)	0.27 (0.18–0.46)	**0.001**
NLR	5.22 (3.22–8.85)	5.03 (3.18–8.35)	8.94 (4.45–19.20)	**0.002**

Quantitative variables are expressed as number (percentage) or median (interquartile range). P value for differences between mild and severe/critical patients. SpO_2_: peripheral capillary oxygen saturation; CRP: C-reactive protein; LDH: lactate dehydrogenase; NLR: neutrophil/lymphocyte ratio.

**Fig 1:**
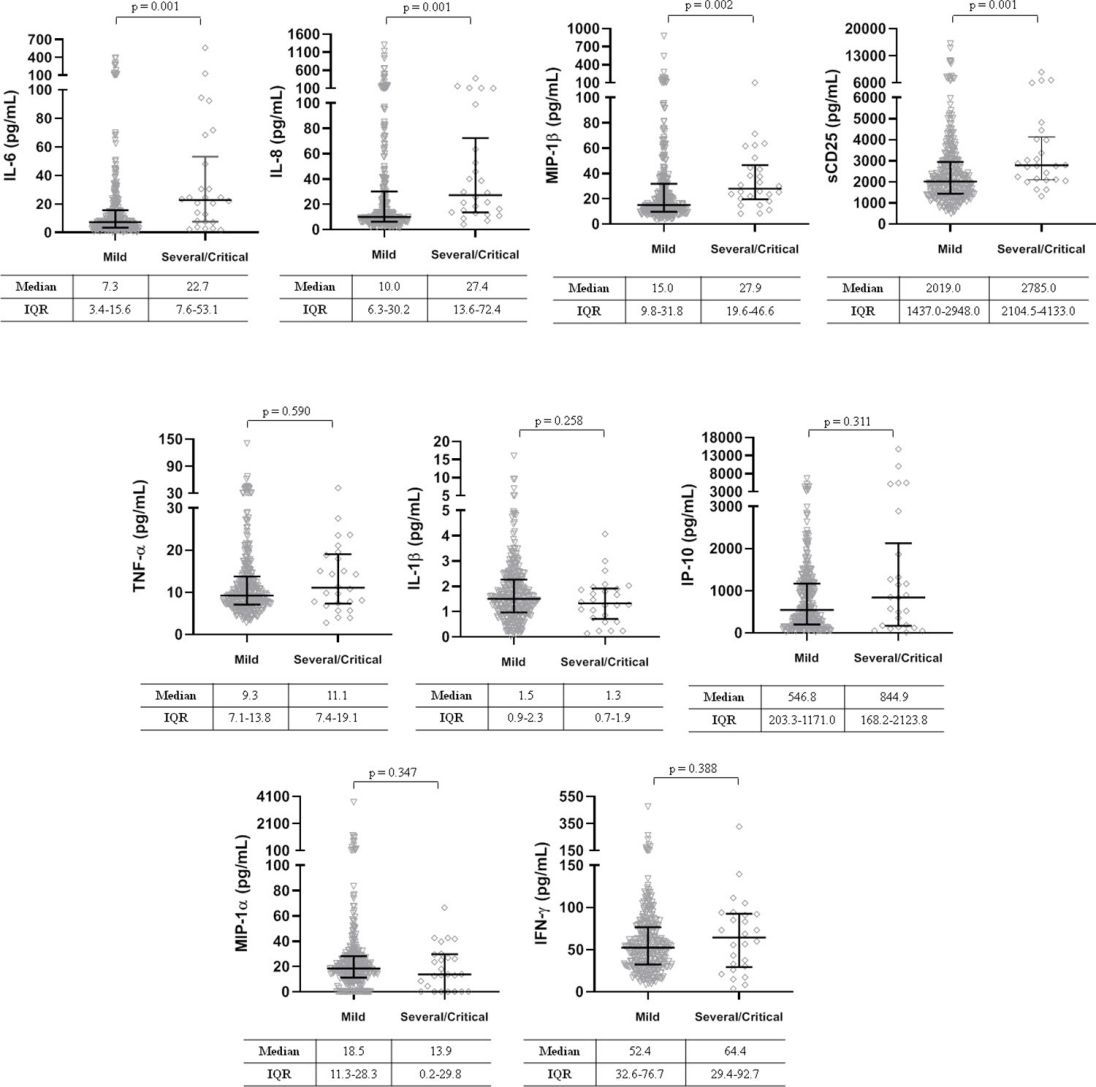
Plasma cytokine concentrations at baseline according to four-point ordinal categories based on oxygen at admission. Mild, oxygen therapy with mask or nasal prongs. Severe/Critical, high-flow oxygen requirement, non-invasive ventilation, mechanical ventilation or extracorporeal membrane oxygenation, and death. IL-6, interleukine-6; IL-8, interleukine-8; MIP-1β, macrophage inflammatory proteins 1β; sCD25, soluble receptor interleukine-2; TNF-α, tumor necrosis factor α; IL-1β, interleukine-1β; IP-10, interferon γ-induced protein 10; MIP-1α, macrophage inflammatory proteins 1α; IFN-γ, interferon-gamma.

Regarding the pro-inflammatory cytokines, we found no differences in plasma concentrations of TNF-α, IL-1β, IP-10, MIP-1α, and IFN-γ between mild and severe/critical patients at admission. By contrast, there were clear differences in plasma IL-6, IL-8, sCD25, and MIP-1β levels, all p ≤0.002 (Fig 1).

### Outcome through the first and second week

**Fig 2:**
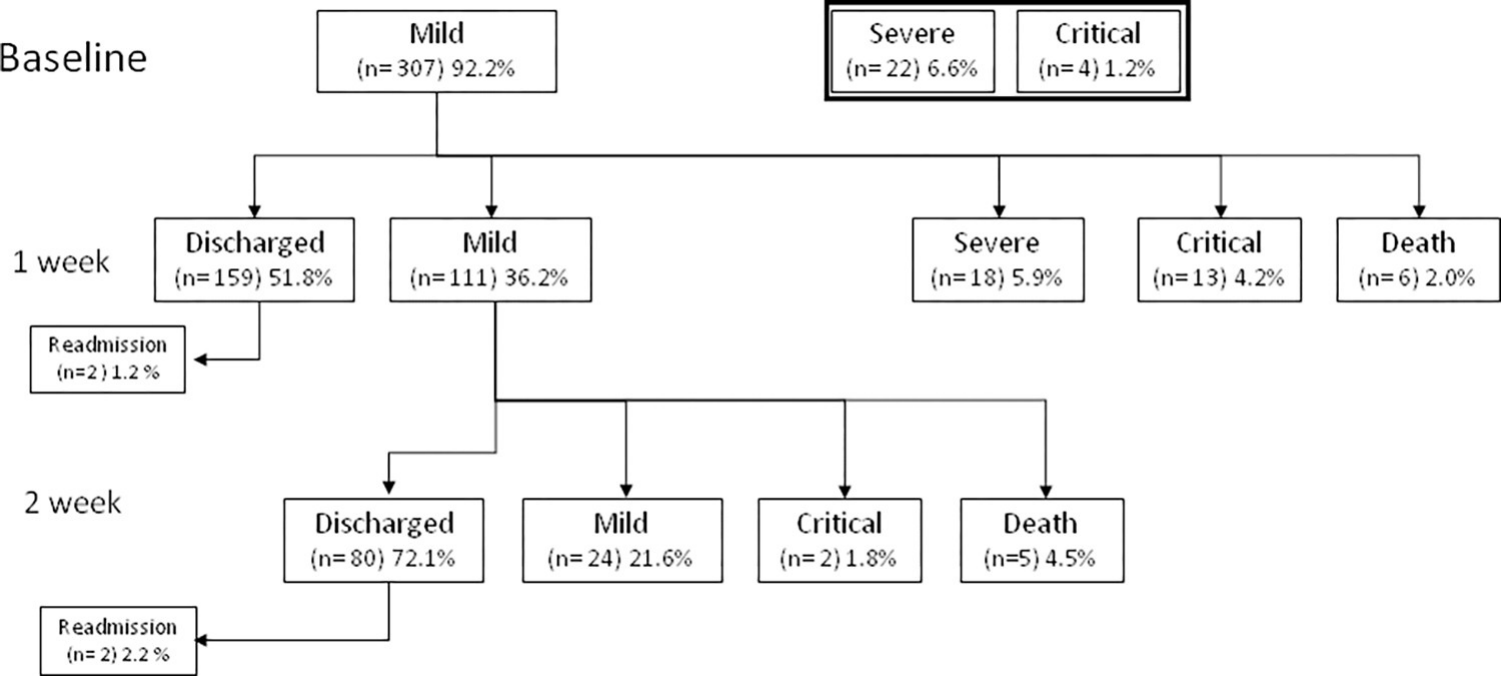
Flow chart of the evolution during hospitalization.

Focusing on the mild group, 159 patients (51.8%) were discharged during the first week after a median of 5 (3–6) days of hospitalization, and 80 (26%) additional patients during the second week after a median of 9 (8–11) days. By contrast, 37 patients (12%) and 7 (2.2%) worsened during the first and second weeks, respectively (Fig 2).

**Fig 3:**
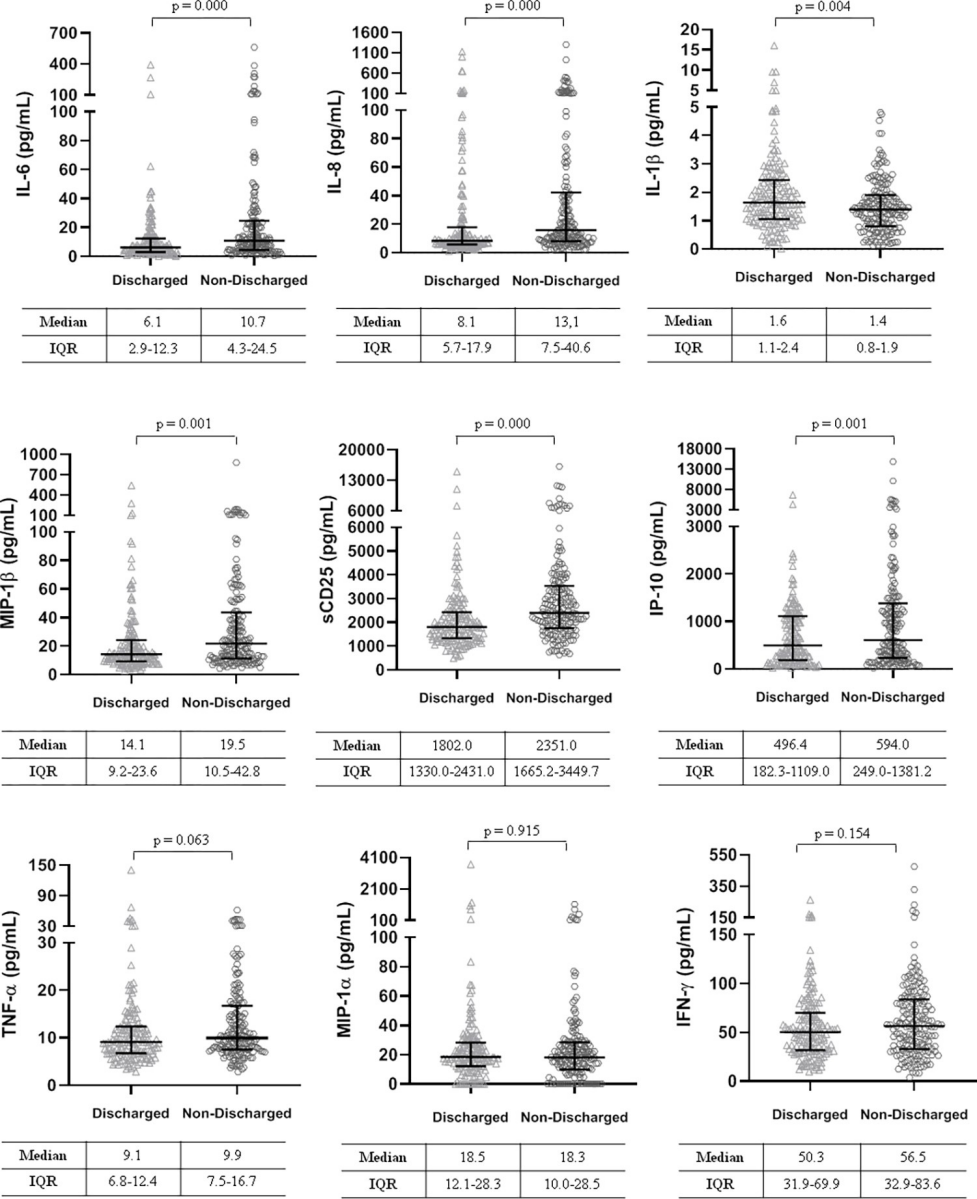
Differences in plasma cytokine concentrations at baseline in mild patients according to whether or not they were discharged during the first week of hospitalization. IL-6, interleukine-6; IL-8, interleukine-8; IL-1β, interleukine-1β; MIP-1β, macrophage inflammatory proteins 1β; sCD25, soluble receptor interleukine-2; IP-10, interferon γ-induced protein 10; TNF-α, tumor necrosis factor α; MIP-1α, macrophage inflammatory proteins 1α; IFN-γ, interferon-gamma.

During the first week, two patients were readmitted due to respiratory failure and reclassified as not discharged. Those patients who discharged or remained stable during the first week, were younger, had a higher SpO_2_, fewer comorbidities (S1 Table) and lower levels of IL-6, IL-8, MIP-1β, sCD25 and IP-10, and slightly higher IL-1β concentrations (all p <0.005) (Fig 3).

**Fig 4:**
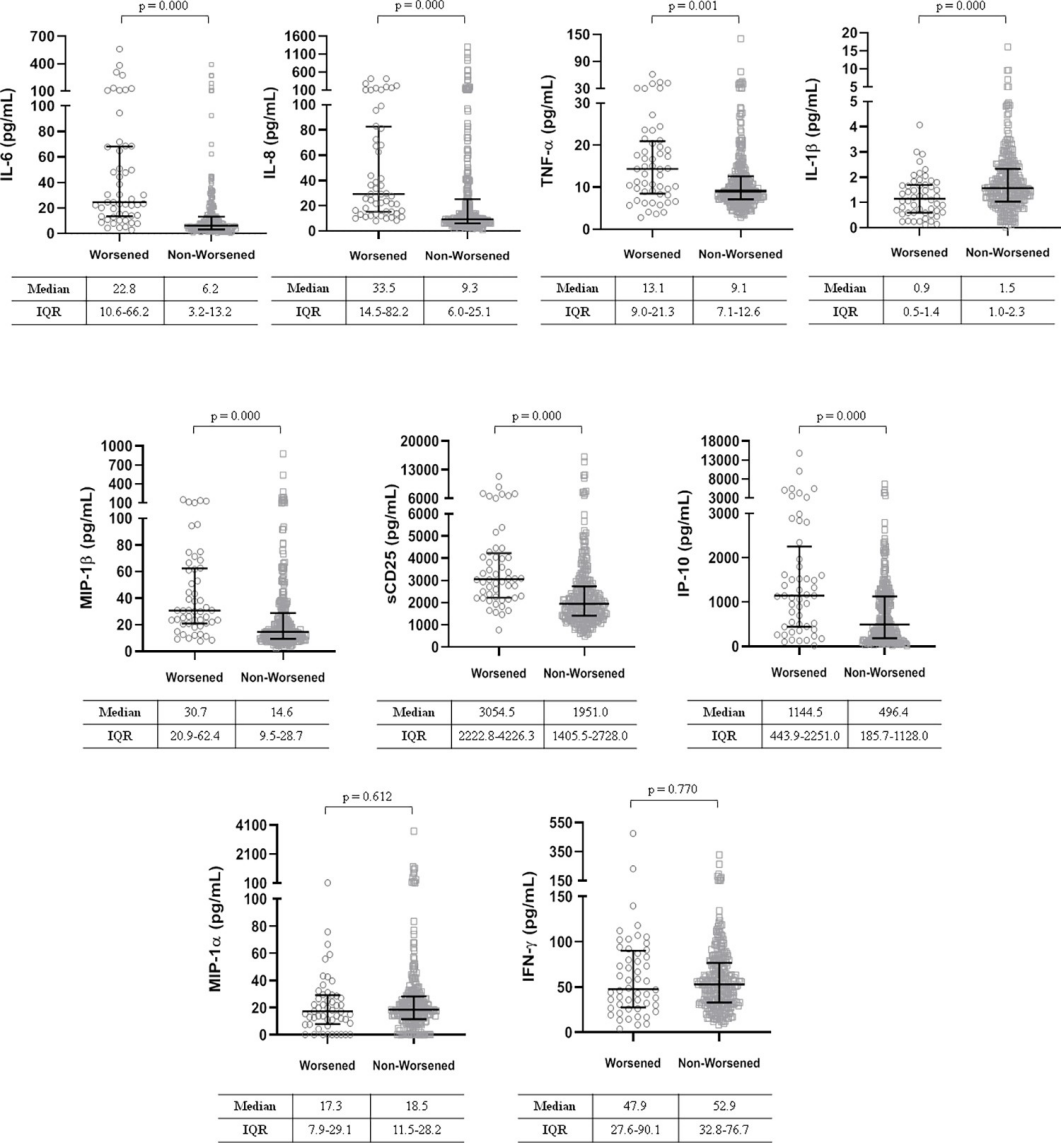
Differences in baseline plasma cytokine concentrations in mild patients according to whether they worsened during the first week of hospitalization or not. IL-6, interleukine-6; IL-8, interleukine-8; TNF-α, tumor necrosis factor α; IL-1β, interleukine-1β; MIP-1β, macrophage inflammatory proteins 1β; sCD25, soluble receptor interleukine-2; IP-10, interferon γ-induced protein 10; MIP-1α, macrophage inflammatory proteins 1α; IFN-γ, interferon-gamma.

However, there were no differences in plasma concentrations of TNF-α, MIP-1α, and IFN-γ. at admission. Compared to patients who were discharged or remained stable during the first week those who worsened were older, predominantly male sex, had a lower SpO_2_ and higher inflammatory markers. Furthermore, the cytokines mentioned above were higher than in those that did not worsen, except for IL-1 β, which showed lower levels, all p <0.001 (Fig 4).

### Predictive models

By ROC curve analyses, no isolated parameter showed enough sensitivity, specificity, PPV, or NPV to differentiate between those patients discharged during the first week or who got worse to be used in the clinical setting with confidence (S2 and S3 Tables).

Therefore, for both situations, we made models with only clinical and routine laboratory parameters easily accessible and added plasma concentrations of different pro-inflammatory cytokines. In all models, we included clinically relevant and statistically significant variables in the previous bivariate analysis, including hospital origin, treatment received, and comorbidities.

In the first case, the parameters associated with discharge during the first week were age <60 years, SpO_2_ ≥ 93%, LDH <337 UI/L, D-dimer <698 ng/mL, NLR <4.76, and treatment with oral corticosteroids during the first days of hospitalization (Table 2A). This model showed an AUC of 0.786 (CI_95_, 0.733–0.839; p <0.0001), a sensitivity of 68.5%, specificity 74.5%, PPV 74.4%, and NPV of 68.9% (S1A Fig). If we include pro-inflammatory cytokine determinations in the model, concentrations of IL-1β ≥1.8 pg/mL, and IP10 <1315 pg/mL were also independently associated with early discharge (Table 2B), achieving an AUC of 0.819 (CI_95_, 0.770−0.867), a sensitivity of 91.7%, specificity 56.6%, PPV 69.3% and NPV of 86.5% (S1A Fig). The internal validation obtained a correct classification rate of 70% for both discharge models.

**Table 2:** Predictive models for early hospital discharge among mild patients at admission.

**A)**	**B (SE)**	**Wald**	**p**	**OR (95% CI)**
SpO_2_ ≥93%	1.00 (0.29)	11.95	0.001	2.74 (1.54–4.85)
Age <60 years	0.89 (0.28)	9.92	0.002	2.44 (1.40–4.27)
Oral corticosteroids	1.203 (0.28)	17.273	<0.001	3.329 (1.88–5.87)
LDH <337 UI/L	1.03 (0.35)	8.51	0.004	2.82 (1.40–5.66)
D-dimers <698 ng/mL	0.53 (0.27)	3.73	0.053	1.71 (0.99–2.96)
NLR <4.76	0.69 (0.28)	5.98	0.014	2.00 (1.14–3.50)
**B)**				
SpO_2_ ≥93%	1.00 (0.29)	11.95	<0.001	3.03 (1.64–5.60)
Age <60 years	0.89 (0.28)	9.92	0.023	1.99 (1.10–3.61)
LDH <337 UI/L	1.203 (0.28)	17.273	0.014	2.47 (1.19–5.10)
Oral corticosteroids	1.03 (0.35)	8.51	<0.001	3.65 (2.00–6.67)
NLR <4.76	0.53 (0.27)	3.73	0.043	1.85 (1.09–3.36)
IL-1β ≥1.8 pg/mL	0.69 (0.28)	5.98	0.001	3.01 (1.63–5.64)
IP-10 <1315 pg/mL	-2.99 (0.46)	41.25	0.045	2.19 (1.08–4.74)

**A)** Model carried out only with clinical parameters and routine laboratory parameters, Nagelkerke R^2^, 0.324. Hosmer-Lemeshow χ^2^, 4.62, df 8; p = 0.796. **B)** Model carried out with clinical parameters, routine laboratory parameters and pro-inflammatory cytokines. Nagelkerke R^2^, 0.402. Hosmer-Lemeshow χ^2^ 9.58, p = 0.296. B, beta coefficient. SE, standard error; OR, odds ratio; SpO_2_, peripheral capillary oxygen saturation; LDH, lactate dehydrogenase; NLR, neutrophil/lymphocyte ratio; IP-10, interferon γ-induced protein 10; IL-1β, interleukine-1β.

Afterward, we performed predictive models to evaluate the parameters associated with worsening during the first week. Among the clinical and routine laboratory parameters, male sex, absence of oral corticosteroids, SpO_2_ <93%, CRP >67 mg/L, NLR ≥5.86, and monocyte count <0.31x10^9^ were independent factors that predicted a poor prognosis, achieving an AUC of 0.903 (CI_95_, 0.852–0.953), with a sensitivity of 97.1%, specificity 68.8%, PPV 30.4%, and NPV of 99.4% (Table 3A and S1 Fig).

**Table 3:** Predictive models for worsening during the first week among mild patients at admission.

**A)**	**B (SE)**	**Wald**	**p**	**OR (95% CI)**
SpO_2_ <93%	1.46 (0.45)	10.15	<0.001	4.32 (1.75–10.62)
Sex (male)	-1.33(0.60)	4.90	0.027	3.84 (1.17–12.5)
No oral corticosteroids	-1.05 (0.46)	4.73	0.030	2.77 (1.11–7.14)
CRP >67 mg/L	1.87 (0.48)	15.07	<0.001	6.54 (2.53–16.88)
NLR ≥5.86	1.96 (0.56)	12.28	<0.001	7.15 (2.38–21.49)
Monocytes <0.315 x 10^9^/L	1.69 (0.49)	11.84	0.001	5.42 (2.07–14.22)
**B)**				
SpO_2_ <93%	1.87 (0.56)	10.92	0.001	6.49 (2.14–19.69)
Sex (male)	1.43 (0.67)	4.53	0.033	4.34 (1.12–16.66)
CRP >67 mg/L	1.23 (0.55)	4.94	0.026	3.44 (1.15–10.22)
NLR ≥5.86	1.84 (0.64)	8.15	0.004	6.31 (1.78–22.40)
Monocytes <0.315 x 10^9^/L	1.93 (0.60)	10.18	0.001	6.94 (2.11–22.85)
IL-8 >9.98 pg/mL	2.18 (0.91)	5.73	0.017	8.88 (1.48–53.13)
MIP-1β >20.4 pg/mL	1.34 (0.64)	4.44	0.037	3.83 (1.08–13.58)
sCD25 >2080 pg/mL	1.36 (0.65)	4.36	0.037	3.91 (1.08–14.08)

**A)** Model carried out only with clinical parameters and routine laboratory parameters, Nagelkerke R^2^, 0.482. Hosmer-Lemeshow χ^2^, 10.25, p = 0.247. **B)** Model carried out with clinical parameters, routine laboratory parameters and pro-inflammatory cytokines. Nagelkerke R^2^, 0.625. Hosmer-Lemeshow χ^2^ 3.62, df 8; p = 0.889. B, beta coefficient; SE, standard error; OR, odds ratio; SpO_2_, peripheral capillary oxygen saturation; CRP, C-reactive protein; NLR, neutrophil/lymphocyte ratio; IL-8, interleukine-8; MIP-1β, macrophage inflammatory proteins 1β; sCD25, interleukine-2 soluble receptor.

The model that included plasma concentrations of pro-inflammatory cytokines (IL-8 >9.95 pg/mL, MIP-1β >20.4 pg/mL, and sCD25 >2080 pg/mL) showed an AUC 0.952 (CI_95_, 0.928–0.976), a sensitivity (98.1%), specificity (82.7%), PPV (51.5%), and NPV (99.6%) (Table 3B and S1 Fig).

If we extend the analysis period to 2 weeks, the same variables will achieve an AUC of 0.891 (CI_95_, 0.846–0.936), with a sensitivity of 95.5%, specificity 79.4%, PPV 36.5%, and NPV of 99.3%. The addition of IL-8, MIP-1β, and sCD25 levels to the model increased the AUC to 0.926 (CI_95_, 0.894–0.957), sensitivity (100%), specificity (74.3%), PPV (38.9%), and NPV (100%). In this case, internal validation obtained a correct classification rate of 90% for both worsening models.

### Monocytes and cytokines

After observing the association between worsening during the first week with peripheral blood monocytopenia, in a subgroup of 19 patients (mild, 10; severe/critical, 9), we analyzed different monocytes subsets and expression of monocyte activation, maturation, and migration markers. No statistically significant differences were found in the percentage of monocyte subsets between mild and severe/critical patients [classical monocytes (CD14+CD16-), 84.5% (75.2–94.4) vs 87.1% (82.8–88.6); intermediate (CD14+CD16+), 3.5% (0.8–5.4) vs 1.7% (0.8–5.1); nonclassical (CD14dimCD16+), 2.4% (0.7–5.2) vs 1.4% (0.4–2.3)] respectively, all p >0.05.

However, severe/critical patients had lower absolute values than mild cases [0.270 x 10^9^/L (0.185–0.460) vs. 0.450 (0.330–0.640), p = 0.001]. Among the activation, maturation and homing markers analyzed, we found a higher expression of CCR2 [MFI, 30450 (IQR, 27142–32883) vs. 27122 (IQR, 23941–29088), p = 0.034] in classical, and TLR2 [MFI, 8912 (IQR, 6892–9518) vs. 6287 (IQR, 5337–7224) p = 0.018] in nonclassical monocyte subsets, respectively, in severe/critical compared to mild patients (S2 Table). Moreover, in the severe/critical group a positive association was observed between the percentage of classical monocytes and plasma levels of IL-8 (ρ = 0.905, p = 0.002) and MIP-1β (ρ = 0.786, p = 0.021) (S2 Fig).

## Discussion

In the absence of fully effective treatment, this pandemic is causing the collapse of many healthcare systems. Having a diagnostic tool to predict the evolution of these patients could be very useful. To date, many studies have evaluated the ability of different parameters to predict a poor prognosis [11, 18, 20–26]. However, none have focused on studying which parameters could reliably predict a favorable outcome for a safe early discharge in hospitalized patients. As in other studies, we found differences in clinical, laboratory parameters, and pro-inflammatory cytokine levels between mild and more severe patients [14, 27–29] (14,28,29); however, although we obtained acceptable data, we consider them not sufficient to discriminate patient outcomes.

A SpO_2_ ≥93%, age ≤60 years and lower LDH, D-dimer, and NLR values were associated with discharge during the first week, with PPV and NPV of 74.4%, and 68.9%, respectively, values too low for clinical application. Among the cytokines analyzed, only lower concentrations of IP-10 and higher concentrations of IL-1β were associated with a better prognosis and discharge during the first week. Although IL-1β is primarily a pro-inflammatory cytokine, it has multiple biological functions, including amplification of the T and B lymphocyte response. This could explain why patients with higher levels of this cytokine and higher lymphocyte counts were discharged early [27]. On the other hand, as do other authors [29–31], we have not observed any relationship between plasma concentrations of IL-1β and worsening mortality [32]. The inclusion of these variables into the model improved predictive capacity by increasing sensitivity and NPV to the detriment of specificity and PPV. Therefore, pro-inflammatory cytokine determinations do not increase clinical utility in predicting early discharge.

On the other hand, the predictive models for worsening during the first week based on admission parameters did not reach sensitivity, specificity and PPV values acceptable for clinical application. However, it should be noted that the NPV reached with clinical data and routine analysis alone was 99.4%. This data could be of great interest in clinical practice since, with a few routine laboratory parameters, it is possible to know which patients are not going to get worse. These patients could be eligible for early discharge or outpatient follow-up, which would mean savings for the health services and help to avoid overcrowding hospitals.

The addition of IL-8, MIP-1β, IP-10, and sCD25 concentrations improved specificity from 68.8% to 82.7% and PPV from 30.4% to 51.5%, with the NPV remaining equal (99.6%). This means that female sex, oral corticosteroids, a SpO_2_ >93%, CRP <67 mg/L, NLR <5.86, and monocyte count >0.31x10^9^ at admission have a predictive value of >99% for not worsening during the first two weeks. Therefore, these patients could be candidates for non-hospital care that could avoid hospital overcrowding.

In addition to variables already known to be associated with worsening, it should be noted that monocytopenia is a predictive factor of worsening during the first week, with a higher expression of CCR2 and TLR2 in classical and non-classical monocytes that are associated with migration and activation, respectively. These results would be in agreement with some studies in which macrophages derived from circulating monocytes have been found in bronchoalveolar samples from patients with severe COVID-19 [33, 34].

The main limitation of the study is the uncontrolled and non-uniform influence of different treatments received by the patients, such as lopinavir/ritonavir, hydroxychloroquine and azithromycin, although it was later found that they were not effective [35]. Likewise, hospital discharge was probably not uniform in all centers or even between the different physicians, nor was the dose of oral steroids administered. [36] Additionally, ferritin levels were not included in the models, as approximately 25% of the patients did not have this determination.

In conclusion, clinical and routine laboratory data at admission strongly predict non-worsening during the first two weeks; therefore, these variables could help to identify those patients who do not need a longer hospitalization and relieve inpatient hospital care. The determination of pro-inflammatory biomarkers moderately improves these predictive capacities.

## Supporting information

S1 Fig A, Predictive models for hospital discharge during the first week in mild patients. B, Predictive models for worsening of clinical status during the first week in patients who were admitted mildly ill. AUC, area under the curve.
(PDF)

S2 Fig Migration and activation marker expressing in monocytes subset in mild and several/critical patients (S/C). (PDF)

S1 Table Baseline characteristics of the mild patients who were discharged and worsened during the first week. Quantitative variables are expressing as number (percentage) or median (interquartile range). P^a^ value for differences between patients who were or not discharged. P^b^ value for differences between patients who who did and did not get wore. SpO_2_, peripheral capillary oxygen saturation; CRP, C-reactive protein; LDH, Lactate dehydrogenase; NLR, neutrophil/lymphocyte ratio.
(PDF)

S2 Table Receiver operating curve (ROC) analyses to evaluate the ability of clinical and laboratory data to predict discharge during the first week. AUC, area under the curve; SE, sensitivity; S, specificity; PPV, positive predictive value; NPV, negative predictive value. SpO_2_, peripheral capillary oxygen saturation; CRP, C-reactive protein; LDH, Lactate dehydrogenase; NLR, neutrophil/lymphocyte ratio; TNF-α; tumor necrosis factor α; IL-6, interleukine-6; IL-8, interleukine-8; IL-1β, interleukine-1β; MIP-1β, macrophage inflammatory proteins 1β; sCD25, soluble receptor interleukine-2; IP-10, interferon γ-induced protein 10.
(PDF)

S3 Table Receiver operating curve (ROC) analyses to evaluate the ability of clinical and laboratory data to predict worse prognosis during the first week. AUC, area under the curve; SE, sensitivity; S, specificity; PPV, positive predictive value; NPV, negative predictive value. SpO_2_, peripheral capillary oxygen saturation; CRP, C-reactive protein; LDH, Lactate dehydrogenase; NLR, neutrophil/lymphocyte ratio; TNF-α; tumor necrosis factor α; IL-6, interleukine-6; IL-8, interleukine-8; IL-1β, interleukine-1β; MIP-1β, macrophage inflammatory proteins 1β; sCD25, soluble receptor interleukine-2; IP-10, interferon γ-induced protein 10.
(PDF)

S4 Table Activation, homing and maturation marker expression in different monocyte subsets. Data are expressed by percentage and interquartile range. Medians fluorescence intensitive (MFI) were calculated in those markets that have a high rate of expression.
(PDF)
