# Correction: Clinical, laboratory data and inflammatory biomarkers at baseline as early discharge predictors in hospitalized SARS-CoV-2 infected patients

**DOI:** 10.1371/journal.pone.0314574

**Published:** 2024-11-21

**Authors:** María Trujillo-Rodriguez, Esperanza Muñoz-Muela, Ana Serna-Gallego, Juan Manuel Praena-Fernández, Alberto Pérez-Gómez, Carmen Gasca-Capote, Joana Vitallé, Joaquim Peraire, Zaira R. Palacios-Baena, Jorge Julio Cabrera, Ezequiel Ruiz-Mateos, Eva Poveda, Luis Eduardo López-Cortés, Anna Rull, Alicia Gutierrez-Valencia, Luis Fernando López-Cortés

There are errors in the author affiliations. The correct affiliations are as follows:

María Trujillo-Rodriguez^1^, Esperanza Muñoz-Muela^1^, Ana Serna-Gallego^1^, Juan Manuel Praena-Fernández^2^, Alberto Pérez-Gómez^1^, Carmen Gasca-Capote^1^, Joana Vitallé^1^, Joaquim Peraire^3,4,5,6^, Zaira R. Palacios-Baena^7,8^, Jorge Julio Cabrera^9^, Ezequiel Ruiz-Mateos^1^, Eva Poveda^10^, Luis Eduardo López-Cortés^7-8^, Anna Rull^3,4,5,6^, Alicia Gutierrez-Valencia^1^, Luis Fernando López-Cortés^1^

**1** Clinical Unit of Infectious Diseases, Microbiology and Preventive Medicine, Institute of Biomedicine of Seville (IBiS), Virgen del Rocío University Hospital, CSIC, University of Seville, Seville, Spain, **2** Department of Biostatistics, Faculty of Medicine, University of Granada, Granada, Spain, **3** Hospital Universitari de Tarragona Joan XXIII (HJ23), Tarragona, Spain, **4** Institut Investigació Sanitària Pere Virgili (IISPV), Tarragona, Spain, **5** CIBER Enfermedades Infecciosas, Instituto de Salud Carlos III, Madrid, Spain, **6** Universitat Rovira i Virgili (URV), Tarragona, Spain, **7** Clinical Unit of Infectious Diseases and Microbiology, Institute of Biomedicine of Seville (IBiS), Virgen del Rocío and Virgen Macarena University Hospitals/CSIC/ University of Seville, Seville, Spain, **8** Clinical Unit of Infectious Diseases and Microbiology, Virgen Macarena University Hospital, Seville, Spain, **9** Microbiology Service, Galicia Sur Health Research Institute (IIS Galicia Sur), Complexo Hospitalario Universitario de Vigo, SERGAS-UVigo, Vigo, Spain, **10** Group of Virology and Pathogenesis, Galicia Sur Health Research Institute (IIS Galicia Sur) Complexo Hospitalario Universitario de Vigo, SERGAS-UVigo, Vigo, Spain.
