# Supplementary material for: Clinical, laboratory data and inflammatory biomarkers at baseline as early discharge predictors in hospitalized SARS-CoV-2 infected patients
Source: PLoS One. 2022 Jul 14;17(7):e0269875. doi: 10.1371/journal.pone.0269875 (PMC9282584; doi:10.1371/journal.pone.0269875)
Supplement: S1 Fig — A, Predictive models for hospital discharge during the first week in mild patients. B, Predictive models for worsening of clinical status during the first week in patients who were admitted mildly ill. AUC, area under the curve. (PDF) [file pone.0269875.s001.pdf]

A

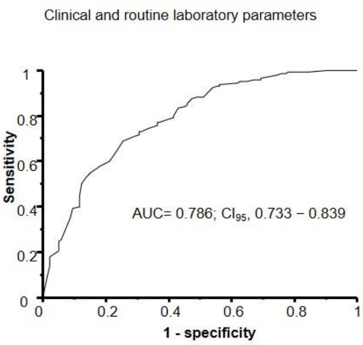

Sensitivity (CI_{95})	Specificity (CI_{95})	PPV (CI_{95})	NPV (CI_{95})
68.5% (63.9-73.0)	74.5% (70.2-78.7)	74.4% (70.1-78.6)	68.9% (64.3-73.4)

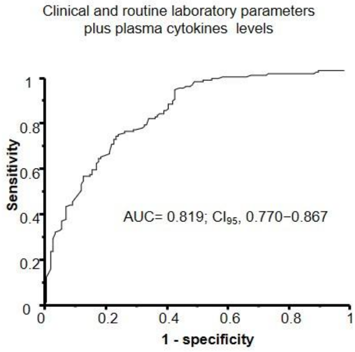

Sensitivity (CI_{95})	Specificity (CI_{95})	PPV (CI_{95})	NPV (CI_{95})
91.7% (88.9-94.4)	56.6% (51.7-61.4)	69.3% (64.7-73.8)	86.5% (83.1-89.8)

B

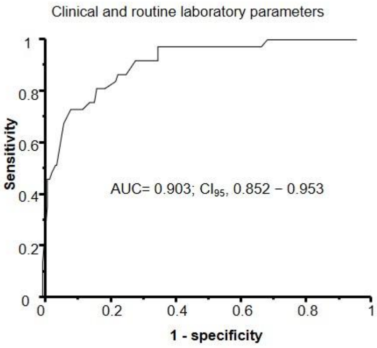

Sensitivity (CI_{95})	Specificity (CI_{95})	PPV (CI_{95})	NPV (CI_{95})
97.1% (95.4-98.7)	68.8% (64.3-73.3)	30.4% (25.8-34.9)	99.4% (98.6-100,1)

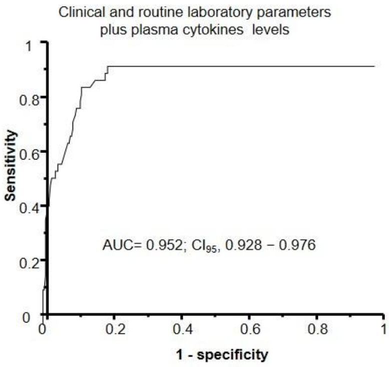

Sensitivity (CI_{95})	Specificity (CI_{95})	PPV (CI_{95})	NPV (CI_{95})
98.1% (96.7-99.4)	82.7% (78.9-86.4)	51.5% (46.6-56.3)	99.6% (98.9-100.2)
