# Supplementary figures and images for: Clinical, laboratory data and inflammatory biomarkers at baseline as early discharge predictors in hospitalized SARS-CoV-2 infected patients
Source: PLoS One. 2022 Jul 14;17(7):e0269875. doi: 10.1371/journal.pone.0269875 (PMC9282584; doi:10.1371/journal.pone.0269875)

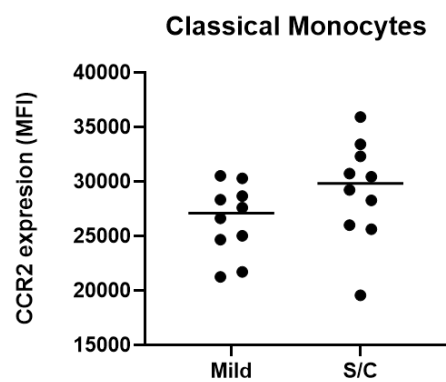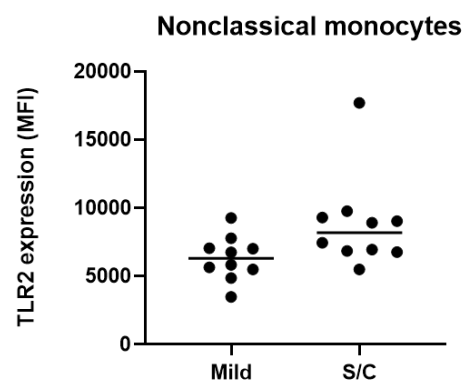

Supplement: S2 Fig — (PDF) [file pone.0269875.s002.pdf]
