# Supplementary material for: Clinical, laboratory data and inflammatory biomarkers at baseline as early discharge predictors in hospitalized SARS-CoV-2 infected patients
Source: PLoS One. 2022 Jul 14;17(7):e0269875. doi: 10.1371/journal.pone.0269875 (PMC9282584; doi:10.1371/journal.pone.0269875)
Supplement: S1 Table — Quantitative variables are expressing as number (percentage) or median (interquartile range). Pa value for differences between patients who were or not discharged. Pb value for differences between patients who who did and did not get wore. SpO2, peripheral capillary oxygen saturation; CRP, C-reactive protein; LDH, Lactate dehydrogenase; NLR, neutrophil/lymphocyte ratio. (PDF) [file pone.0269875.s003.pdf]

	Discharged (n=157)	Non-discharged (n=150)	P[a]	Non-Worsened (n= 281)	Worsened (n=37)	P[b]
Age (years)	57 (42-70)	68 (55-78)	0.000	61 (47-74)	67 (55-78)	0.022
Male sex.	95 (60.5)	98 (65.3)	0.383	160 (59.3)	33 (89.2)	0.000
Comorbidities	104 (66.2)	117 (78.0)	0.022	191 (70.7)	30 (81.1)	0.190
Diabetes mellitus	30 (19.1)	35 (23.3)	0.523	53 (19.6)	12 (32.4)	0.079
Hypertension	53 (33.8)	80 (53.3)	0.002	111 (41.1)	22 (59.5)	0.036
Heart disease	18 (11.5)	35 (23.3)	0.011	46 (17)	7 (18.9)	0.790
Chronic pulmonary disease	16 (10.2)	19 (12.7)	0.605	29 (10.7)	6 (16.2)	0.333
Cancer	5 (3.2)	14 (9.3)	0.034	14 (5.2)	5 (13.5)	0.050
Symptoms on admission						
Cough	95 (60.5)	106 (70.7)	0.201	179 (66.3)	22 (59.5)	0.370
Fever	94 (59.9)	90 (60.0)	0.722	160 (59.)	24 (64.9)	0.585
Dyspnea	64 (40.8)	77 (51.3)	0.366	126 (46.7)	15 (40.5)	0.603
Diarrhea	40 (25.5)	36 (24.0)	0.572	70 (25.9)	6 (16.2)	0.191
Arthromyalgia	37 (23.6)	29 (19.3)	0.252	58 (21.5)	8 (21.6)	1.000
SpO_2 (%)	96 (94-97)	94 (91-96)	0.000	95 (93-97)	91 (89-95)	0.000
Onset of symptoms until sampling (days)	8 (6-11)	8 (5-12)	0.569	8 (5-12)	7 (5-9)	0.013
CRP (mg/L)	18 (5-54)	35 (8-101)	0.007	20 (5-64)	96 (29-173)	0.000
LDH (UI/L)	256 (218-309)	280 (232-375)	0.004	259 (221-315)	338 (274-448)	0.000
D-dimer (ng/mL)	543 (385-960)	787 (414-1263)	0.004	590 (390-1030)	836 (450-1638)	0.055
Ferritin (ng/mL)	390 (205-735)	526 (232-1222)	0.033	397 (198-837)	1339 (429-1623)	0.000
Lymphocytes (x 10^9/L)	1.13 (0.84-1.60)	0.98 (0.72-1.40)	0.006	1.13 (0.79-1.52)	0.81 (0.54-0.90)	0.000
Neutrophils (x 10^9/L)	5.50 (3.64-7.30)	6.60 (4.53-8.21)	0.000	5.8 (3.84-7.60)	7.42 (4.72-9.49)	0.010
Monocytes (x 10^9/L)	0.45 (0.33-0.61)	0.45 (0.32-0.64)	0.795	0.46 (0.29-0.78)	0.36 (0.27-0.65)	0.106
NLR	4.31 (2.8-6.7)	6.15 (3.9-9.9)	0.000	4.69 (2.99-7.87)	8.06 (5.92-14.51)	0.000
Hospitalized days	5 (3-6)	11 (9-19)	0.000	7 (5-10)	17 (9-37)	0.000
Treatment during first week						
Oral dexamethasone	83 (52.9)	48 (32.0)	0.034	73 (27.0)	5 (13.5)	0.006
Bolus of corticosteroids	41 (26.1)	31 (20.7)	0.261	65 (24.1)	7 (28.8)	0.488
Remdesivir	32 (20.4)	19 (12.7)	0.070	49 (18.1)	2 (5.4)	0.051
