# Supplementary material for: Clinical, laboratory data and inflammatory biomarkers at baseline as early discharge predictors in hospitalized SARS-CoV-2 infected patients
Source: PLoS One. 2022 Jul 14;17(7):e0269875. doi: 10.1371/journal.pone.0269875 (PMC9282584; doi:10.1371/journal.pone.0269875)
Supplement: S2 Table — AUC, area under the curve; SE, sensitivity; S, specificity; PPV, positive predictive value; NPV, negative predictive value. SpO2, peripheral capillary oxygen saturation; CRP, C-reactive protein; LDH, Lactate dehydrogenase; NLR, neutrophil/lymphocyte ratio; TNF-α; tumor necrosis factor α; IL-6, interleukine-6; IL-8, interleukine-8; IL-1β, interleukine-1β; MIP-1β, macrophage inflammatory proteins 1β; sCD25, soluble receptor interleukine-2; IP-10, interferon γ-induced protein 10. (PDF) [file pone.0269875.s004.pdf]

Variables	Cut-off point	AUC (95% CI)	SE	S	PPV	NPV
SpO_2 (%)	93	0.689 (0.630-0.749)	88.7	48.3	61.0	82,4
CRP (mg/L)	55	0.606 (0.545-0.667)	81.1	39.1	55.8	69.4
Ferritin (ng/mL)	529	0.574 (0.503-0.645)	54.7	63.2	37.7	60.4
D-dimer (ng/mL)	698	0.608 (0.543-0.674)	60.4	60.9	41.6	62.7
LDH (UI/L)	337	0.621 (0.560-0.681)	84.3	40.8	58.0	74.4
Neutrophiles ($x10^9$/L)	6.89	0.639 (0.508-0.698)	72.3	48.3	49.8	65.6
Lymphocytes ($x10^9$/L)	0.80	0.612 (0.552-0.672)	70.4	49.4	48.5	64.7
NLR	4.76	0.665 (0.607-0.723)	60.4	69.5	41.6	65.8
TNF-α (pg/mL)	16.4	0.568 (0.506-0.629)	86.8	40.8	59.7	77.2
IL-6 (pg/mL)	7.6	0.645 (0.586-0.704)	64.2	65.5	44.2	66.7
IL-8 (pg/mL)	9.6	0.646 (0.586-0.705)	60.4	67.8	41.6	67.4
IL-1β (pg/mL)	1.8	0.599 (0.538-0.660)	44.7	73.6	30.7	59.3
MIP-1β (pg/mL)	20.8	0.627 (0.567-0.687)	73.0	52.3	50.2	67.9
sCD25 (pg/mL)	2064	0.670 (0.612-0.728)	65.4	66.1	45.0	67.6
IP-10 (pg/mL)	1315	0.577 (0.516-0.638)	88.1	27.6	60.6	71.6
