# Supplementary material for: Clinical, laboratory data and inflammatory biomarkers at baseline as early discharge predictors in hospitalized SARS-CoV-2 infected patients
Source: PLoS One. 2022 Jul 14;17(7):e0269875. doi: 10.1371/journal.pone.0269875 (PMC9282584; doi:10.1371/journal.pone.0269875)
Supplement: S3 Table — AUC, area under the curve; SE, sensitivity; S, specificity; PPV, positive predictive value; NPV, negative predictive value. SpO2, peripheral capillary oxygen saturation; CRP, C-reactive protein; LDH, Lactate dehydrogenase; NLR, neutrophil/lymphocyte ratio; TNF-α; tumor necrosis factor α; IL-6, interleukine-6; IL-8, interleukine-8; IL-1β, interleukine-1β; MIP-1β, macrophage inflammatory proteins 1β; sCD25, soluble receptor interleukine-2; IP-10, interferon γ-induced protein 10. (PDF) [file pone.0269875.s005.pdf]

Variables	Cut-off point	AUC (95% CI)	SE	S	PPV	NPV
SpO_2 (%)	93	0.787 (0.715-0.859)	71.2	77.2	36.6	93.5
CRP (mg/L)	67	0.745 (0.674-0.816)	65.4	76.5	34.0	92.3
Ferritin (ng/mL)	747	0.725 (0.644-0.807)	48.1	77.9	28.7	89.0
D-dimer (ng/mL)	727	0.645 (0.566-0.725)	67.3	61.9	24.6	91.1
LDH (UI/L)	328	0.738 (0.658-0.818)	61.5	79.0	35.2	92.9
Neutrophiles (x10^9/L)	7.18	0.647 (0.561-0.734)	57.7	66.9	24.4	89.5
Lymphocytes (x10^9/L)	0.87	0.711 (0.636-0.785)	71.2	69.4	30.1	92.9
Monocytes (x10^9/L)	0.31	0.628 (0.537-0.718)	50.0	79.7	31.3	89.6
NLR	5.86	0.628 (0.537-0.718)	50.0	79.7	31.3	89.6
TNF-α (pg/mL)	12.8	0.725 (0.655-0.795)	78.8	63.0	28.3	94.1
IL-6 (pg/mL)	13.2	0.650 (0.560-0.740)	55.8	76.2	30.2	76.2
IL-8 (pg/mL)	9.9	0.827 (0.770-0.884)	76.9	77.2	38.5	94.8
IL-1β (pg/mL)	0.85	0.767 (0.714-0.820)	96.2	54.4	28.1	98.7
MIP-1β (pg/mL)	20.4	0.650 (0.569-0.730)	38.5	83.6	30.0	88.0
sCD25 (pg/mL)	2080	0.787 (0.715-0.859)	71.2	77.2	36.6	93.5
IP-10 (pg/mL)	1063	0.745 (0.674-0.816)	65.4	76.5	34.0	92.3
