# Supplementary material for: Clinical, laboratory data and inflammatory biomarkers at baseline as early discharge predictors in hospitalized SARS-CoV-2 infected patients
Source: PLoS One. 2022 Jul 14;17(7):e0269875. doi: 10.1371/journal.pone.0269875 (PMC9282584; doi:10.1371/journal.pone.0269875)
Supplement: S4 Table — Data are expressed by percentage and interquartile range. Medians fluorescence intensitive (MFI) were calculated in those markets that have a high rate of expression. (PDF) [file pone.0269875.s006.pdf]

Monocyte markers	Classical			Intermediate			NonClassical		
	**Mild**	**S/C**	**p**	**Mild**	**S/C**	**p**	**Mild**	**S/C**	**p**
CCR2	96.2 (91.7-97.5)	93.6 (90.2-96.9)	0.497	53.0 (38.0-70.5)	52.4 (21.5-71.2)	0.661	1.4 (0.0-3.1)	0.7 (0.1-3.1)	0.842
CCR2 (MFI)	27122 (23941-29088)	30450 (27142-33340)	**0.035**	29307 (18378-35336)	36547 (31924-37814)	0.079	10464 (8161-14439)	13989 (11908-21666)	0.121
CCR5	1.65 (1.11-3.62)	1.32 (1.1-2.2)	0.447	10.8 (10.0-23.8)	11.9 (7.9-18.1)	0.780	6.3 (1.8-9.2)	4.79 (1.8-21.1)	0.842
CD11b	94.5 (93.1-95.5)	94.1 (91.5-94.5)	0.356	80.2 (71.9-88.4)	69.3 (52.7-87.2)	0.356	7.2 (2.7-10.6)	5.6 (4.0-29.6	0.842
CD11b (MFI)	3633 (2847-3834)	3018(2824-3325)	0.133	3215 (2887-3625)	2802 (2472-3111)	0.095	2236 (2141-3918)	2110 (1668-2336)	0.113
CD40	5.0 (2.2-9.9)	5.6 (1.8-11.7)	0.842	39.4 (23.4-71.6)	49.7 (29.7-64.8)	0.905	16.1 (12.7-22.5)	16 (11.1-51.3)	0.720
CD49d	0.6 0.4-2.0)(	1.0 (0.4-1.7)	0.905	5.0 (2.1-9.7)	3.8 (2.1-7.7)	0.780	0.1 (0.0-0.0)	0.3 (0.0-1.0)	0.315
CX3CR1	0.1 (0.0-0.2)	0.07 (0.04-0.15)	0.243	1.8 (0.2-5.7)	2.1 (0.8-75.8)	0.842	2.4 (0.6-3.5)	5.3 (0.8-15.3)	0.133
Tissue Factor	0.6 (0.2-1.4)	0.7 (0.5-0.9)	0.780	10.2 (8.5-29.0)	13.5 (12.4-19.4)	0.243	14.8 (7.9-25.0)	8.2 (6.8-18.7)	0.400
TLR2	98.9 (98.5-99.7)	99.4 (99.1-99.7)	0.156	98.9 (97.9-99.7)	99.5 (98.1-99.9)	0.497	44.5 (23.4-95.8)	93.3 (55.0-97.9)	0.065
TLR2 (MFI)	6175 (5364-8169)	6704(5809-7175)	0780	8444 (7953-10183)	8792 (8521-9956)	0.549	6287 (5337-7224)	8912 (6892-9519)	**0.017**
TLR4	0.6 (0.3-1.0)	0.5 (0.3-0.8)	0.780	2.2 (1.2-4.0)	1.9 (0.9-2.0)	0.243	0.5 (0.0-1.2)	0.3 (0.0-1.0)	0.661
